# Taxonomic review of the mantidfly genus *Nolima* Navás (Neuroptera, Mantispidae, Calomantispinae)

**DOI:** 10.3897/zookeys.853.30317

**Published:** 2019-06-06

**Authors:** Daniel Reynoso-Velasco, Atilano Contreras-Ramos

**Affiliations:** 1 Red de Biodiversidad y Sistemática, Instituto de Ecología, AC (INECOL), Carretera Antigua a Coatepec Núm. 351, El Haya, 91070, Xalapa, Veracruz, México Red de Biodiversidad y Sistemática, Instituto de Ecología Xalapa Mexico; 2 Instituto de Biología-UNAM, Departamento de Zoología, 04510 Ciudad de México, México Universidad Nacional Autónoma de México Mexico City Mexico

**Keywords:** Lacewings, mantispids, New World, new species, taxonomy

## Abstract

The mantidfly genus *Nolima* Navás, 1914 (Neuroptera, Mantispidae, Calomantispinae) is herein revised. *Nolima* is endemic to the New World, ranging from the southwestern United States south to Costa Rica. *Nolimainfensa* Navás, *N.pinal* Rehn, and *N.victor* Navás are redescribed, while the new species *Nolimacostaricensis* Reynoso & Contreras, **sp. nov.** is described from Costa Rica. The species *N.dine* Rehn and *N.kantsi* Rehn are synonymized with *N.pinal*. Additionally, the species *N.praeliator* Navás and *N.pugnax* Navás are synonymized with *N.victor*, for which a lectotype is designated. New distribution records are provided from Guatemala and Honduras for *Nolimainfensa*, the state of Nevada in western United States for *N.pinal*, and the state of Puebla in central Mexico for *N.victor*. An illustrated key and a distribution map are presented.

## Introduction

**Figure 1.**
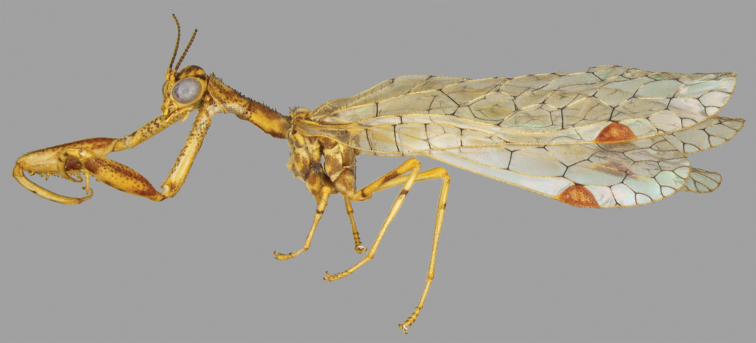
Habitus of a male of *Nolimavictor* (abdomen removed).

Mantidflies, mantid lacewings, or mantispids (Mantispidae) are distinctive within the Neuroptera because of their raptorial forelegs (Fig. 1), which are convergent in some Rhachiberothidae. The taxonomic knowledge of the New World mantispid fauna is still fragmentary (Ohl 2005). Noteworthy previous contributions are a genus-level revision by Penny (1982) and the works by Hoffman (1992, 2002) on the subfamily Mantispinae. In the Nearctic, Rehn (1939) revised the genus *Plega* Navás. In the Neotropics, Penny (1982) and Penny and da Costa (1983) studied the fauna of Brazil. Most recently, Reynoso-Velasco and Contreras-Ramos (2008) studied the Mexican fauna of Mantispidae, Machado and Rafael (2010) treated the Brazilian species previously placed in *Mantispa* Illiger, and Ardila-Camacho and García (2015) and Ardila-Camacho et al. (2018) studied the Mantispidae from Colombia and Panama. Additionally, Hoffman et al. (2017) treated the Antillean fauna of Mantispidae.

**Figure 2.**
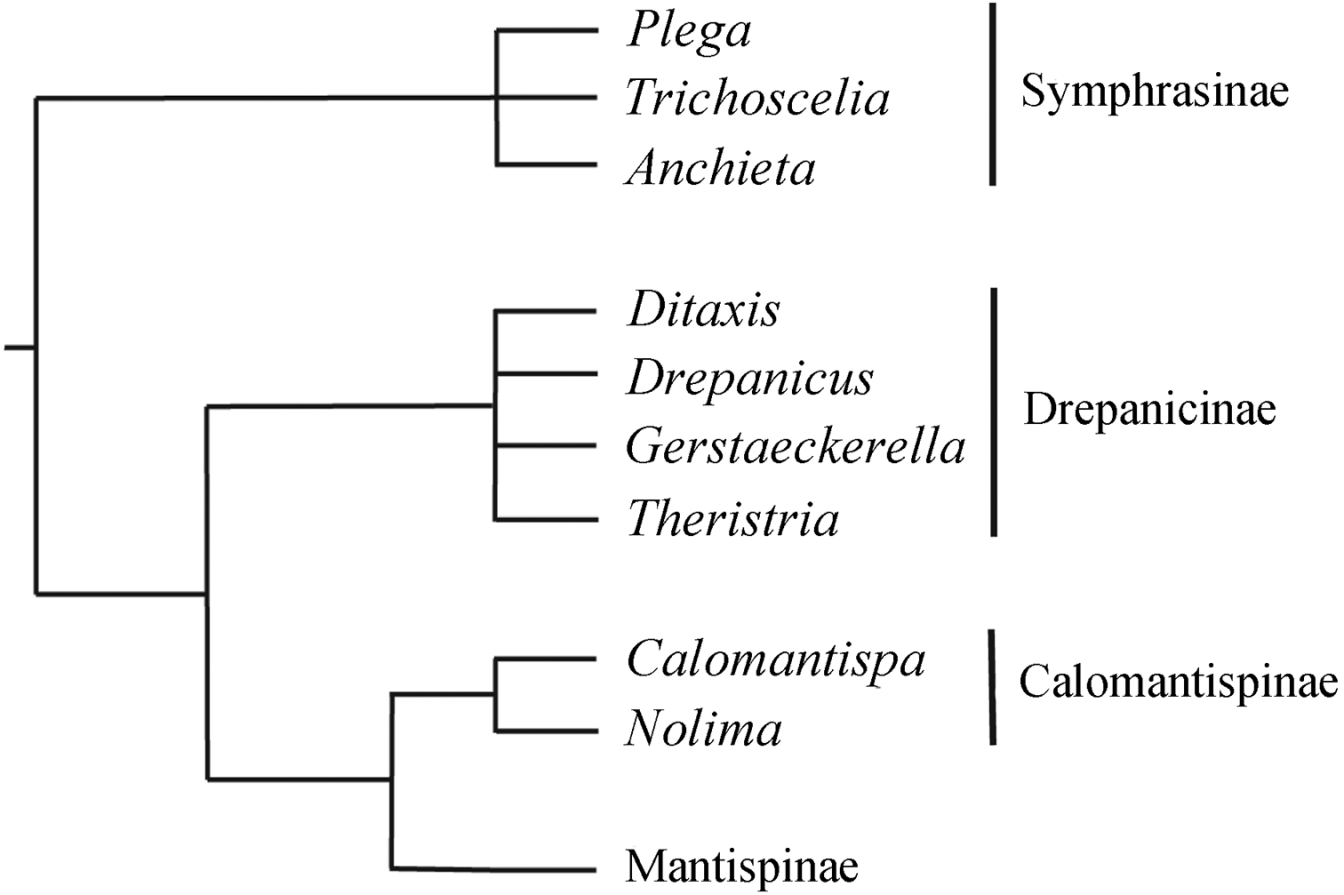
Phylogenetic relationships among subfamilies in Mantispidae (modified from Lambkin 1986a).

Four subfamilies of mantidflies are currently recognized: Calomantispinae, Drepanicinae, Mantispinae, and Symphrasinae (Lambkin 1986a, b, Ohl 2004). *Calomantispa* Banks and *Nolima* Navás are generally taken to constitute the subfamily Calomantispinae. As originally proposed by Lambkin (1986a), the subfamily Symphrasinae was the sister group of the clade including Calomantispinae, Drepanicinae, and Mantispinae (Fig. 2). Willman (1990) found the same topology in his study on the phylogenetic relationships between Rhachiberothinae and Mantispidae. Lambkin (1986a) stated that Calomantispinae (*Calomantispa* + *Nolima*) was more closely related to Mantispinae than to Drepanicinae; this scheme was supported in the study by Liu et al. (2015), where the authors included information from DNA sequences and morphological characters. A recent study on the evolution of Neuropterida based on genomic data (Winterton et al. 2018) recovered a paraphyletic Mantispidae, where Calomantispinae was placed sister to Drepanicinae, together forming a clade sister to Mantispinae.

This study consists of the taxonomic revision of the New World genus *Nolima* Navás, which previously included seven nominal species and is the sole representative of the subfamily Calomantispinae in this part of the world. The distribution of the species in this genus ranges from southern United States south to Costa Rica in Central America. The original descriptions of the species in *Nolima* were mainly based on the pigmentation pattern on the head and prothorax. We noticed that those patterns were not consistent and of little help for species identification; for that reason, we decided to study the group and explore other characters (e.g., male genital structures) to better circumscribe the different species.

## Materials and methods

### Specimen sources

The specimens examined during this study, including species from other genera of Mantispidae (Table 1) that were used to establish the diagnostic features of the genus *Nolima*, were obtained through museum loans. The majority of type specimens were studied in situ at their depository collections. Status and validity of the species names were corroborated on the Neuropterida Species of the World Catalog (Oswald 2018). Information given in brackets [] did not appear on the specimen labels nor was no provided in publications, but was inferred from available data or represents corrections to misspellings on the labels. Specimens were obtained on loan from the following collections:

**ANIC** Australian National Insect Collection (Canberra, Australia)

**NHMUK** Natural History Museum (London, England)

**CAS** California Academy of Sciences (San Francisco, United States)

**CNIN** Colección Nacional de Insectos, Universidad Nacional Autónoma de México (Mexico City, Mexico)

**EBCH** Estación de Biología Chamela, Universidad Nacional Autónoma de México, (San Patricio, Mexico)

**ECOSUR** El Colegio de la Frontera Sur (San Cristóbal de las Casas, Mexico)

**FSCA** Florida State Collection of Arthropods (Gainesville, United States)

**INBIO** Instituto Nacional de Biodiversidad (Santo Domingo de Heredia, Costa Rica)

**MCZ** Museum of Comparative Zoology, Harvard University (Cambridge, United States)

**MNHN** Muséum national d’Histoire naturelle (Paris, France)

**QDPI** Queensland Department of Primary Industries (Brisbane, Australia)

**SDMC** San Diego Natural History Museum (San Diego, United States)

**SRSU** Sul Ross State University (Alpine, United States)

**TAMU** Texas A&M University (College Station, United States)

**USNM** United States National Museum of Natural History (Washington DC, United States)

**ZMB** Museum für Naturkunde, Humboldt-Universität (Berlin, Germany)

**Table 1.** Comparative taxa examined to establish diagnostic features of the genus *Nolima* Navás.

Taxon	Distribution	Sex / Repository
Calomantispinae		
*Calomantispapicta* Stitz	Australia: Australian Capital Territory: Canberra.	1♂, 1♀ / ANIC
*Calomantispaspectabilis* Banks	Australia: Queensland: Herberton.	1♂ / ANIC
*Calomantispavenusta* Lambkin	Australia: Australian Capital Territory: Mount Gingera.	1♀ / ANIC
	Australia: Australian Capital Territory: Lee’s Spring.	1♂ / ANIC
	Australia: New South Wales: South Black Range.	1♀ / QDPI
Drepanicinae		
*Drepanicuschrysopinus* Brauer	Chile: Los Ríos: Valdivia.	1♂, 1♀ / CAS
*Gerstaeckerellachilensis* (Hagen)	Chile: Metropolitana de Santiago: Til-Til, Santa Maria.	1♂ / CAS
*Theristriastigma* (Esben-Petersen)	Australia: Queensland: West Claudie River.	1♀ / QDPI
*Theristriastoreyi* Lambkin	Australia: Queensland: Kennedy River.	1♂ / QDPI
Mantispinae		
*Climaciellabrunnea* (Say)	Mexico: Veracruz: San Andrés Tuxtla.	1♀ / CNIN
	Mexico: Veracruz: Santiago Tuxtla.	1♂ / CNIN
*Dicromantispainterrupta* (Say)	Mexico: Jalisco: Estación de Biología Chamela.	1♂ / CNIN
*Dicromantispasayi* (Banks)	Mexico: Chihuahua: El Jaquex.	1♀ / CNIN
*Zeugomantispavirescens* (Rambur)	Mexico: San Luis Potosí: El Limoncito.	1♂ / CNIN
Symphrasinae		
*Plegadactylota* Rehn	México: Baja California Sur.	1♂ / CNIN
*Trichoscelia* sp. 1	Mexico: Sonora: Cerro Verde.	1♂ / CNIN

### Dissecting techniques and illustration

Pinned specimens were placed in an airtight chamber with a solution of water and phenol for rehydration for approximately 24 hours. The abdomen of males was dissected and placed in 10% KOH for approximately 10 hours at room temperature, then rinsed in distilled water. The abdomen of each females was treated similarly, except that it was stained with Chlorazol Black E (in ethanol) to enhance contrast of the internal structures. The dye was injected with a syringe into the abdominal cavity for approximately 10 seconds, then the dissected abdomen was transferred to 70% ethanol and the dye was rinsed out. For observation, the abdomen was placed in a Petri dish with glycerin. A Zeiss Stemi SV11 stereomicroscope with 10× eyepieces and 1.0× and 2.5× main objectives (with a zoom magnifying range of 0.6–6.6×) was used for morphological examination. After examination, the dissected abdomens were stored in genitalia microvials with glycerin and pinned under the corresponding specimen. Pencil drawings were elaborated with a camera lucida attached to the stereomicroscope, which were later inked and scanned. Digital images were obtained by use of a Nikon SMZ25 stereomicroscope coupled with the Nikon NIS-Elements Imaging Software. Final figures were prepared with Photoshop CS5 (Adobe Systems Inc., San Jose, California).

### Morphological terminology

**Figure 3.**
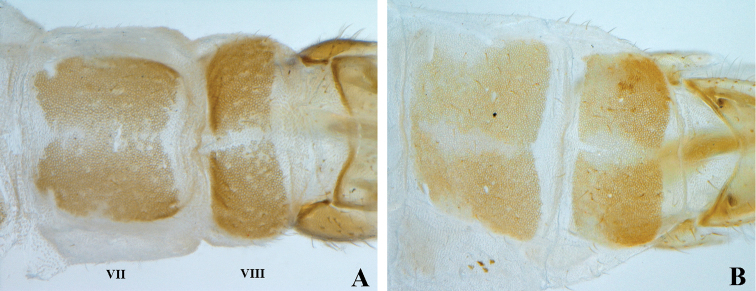
Abdominal terga VII–VIII of *Nolima* species. **A***Nolimainfensa***B***Nolimavictor*.

This study mainly follows Lambkin (1986a, b). In males, the abdominal terga and sterna present sclerotized circular (Fig. 3A) or polygonal structures (Fig. 3B) that we consider are cuticular depressions, but lack a formal name. Such cuticular condition is a reliable diagnostic feature and in the text is simply referred to as circular or polygonal structures. We consider these structures not to be homologous to the abdominal pores of Mantispinae. The term gonarcal membrane is used for the membrane located between the base of the gonarcus, the ninth gonocoxite, and the pseudopenis of males. Females present a protuberant ovoid sclerotized structure associated to the spermatheca that may be a gland, so it is referred to as an accessory gland.

### Diagnostic characters

External and internal structures of males and females where evaluated to serve as potential diagnostic features. The morphology of the female genitalia was found to be conserved and similar among the specimens examined. For that reason, only a generic description of the structures is provided. Attributes related to the presence and position of bristle-bearing chalazae and the pigmentation pattern on the forelegs of both sexes were found to be informative, although the most reliable diagnostic features were related to characteristics of the male abdominal cuticle and genital structures.

## Systematics

The genus *Nolima* was erected (Navás 1914) for the species *Nolimavictor* and *N.praeliator*, both from the Mexican state of Guerrero. In the same work, Navás (1914) created the tribe Nolimini to place the newly created genus. Later, *N.infensa* was described from Costa Rica (Navás 1924) and the species *N.dine* (Arizona), *N.kantsi* (Texas), and *N.pinal* (Arizona) were described from southwestern United States (Rehn 1939). Navás (1914) also created the genus *Bellarminus*, with the Central American (Guatemala) *Bellarminuspugnax* as the type species. Thereafter, Penny (1982) synonymized the genus *Bellarminus* under *Nolima*, so that *N.pugnax* (Navás) became the seventh nominal species in *Nolima*.

As proposed by Lambkin (1986a), the genera *Nolima* and *Calomantispa*, this latter endemic to Australia (Ohl 2004), are included in the subfamily Calomantispinae. This relationship was based on the unique shared characteristics of the bifid foretarsal claws, as well as the scoop-like ninth sternum of the male, which extends posteriorly beyond the ectoprocts. We consider *Nolima* to be monophyletic based on the forewing with a short subcostal space (long in *Calomantispa*), the female spermatheca with a distal accessory gland (proximal in *Calomantispa*), and the male mediuncus with the apex strongly produced posteriorly (not produced or only slightly produced in *Calomantispa*).

### Genus Nolima

Taxon classificationAnimaliaNeuropteraMantispidae

Navás, 1914

Nolima Navás, 1914: 100–101 (original description, gender: feminine, etymology: anagram of Molina, type species by original designation: Nolimavictor); Rehn 1939: 238, 256 (key, description); Acker 1960: 29, 92–93 (species list, illustrations); Penny 1977: 36 (species list); MacLeod and Redborg 1982: 39 (biology); Penny 1982: 212–213 (synonymy); Lambkin 1986a: 3, 9, 15–20, 28, 30, 84 (species list, systematics); Willman 1990: 261 (systematics); Oswald and Penny 1991: 45 (genera list); Henry et al. 1992: 439, 449 (key, species list); Hoffman 2002: 251–252 (key, species list); Ohl 2004: 157–158 (species list); Ohl 2005: 80 (distribution); Reynoso-Velasco and Contreras-Ramos 2008: 704–705, 708 (key, species list); Reynoso-Velasco and Contreras-Ramos 2009: 710 (species list); Reynoso-Velasco and Contreras-Ramos 2010: 270 (distribution); Cancino-López et al. 2015: 201–202, 205 (genera list, species list, systematics); Liu et al. 2015: 184, 194, 201, 204 (genera list, systematics, distribution).
Bellarminus Navás, 1914: 102–103 (original description, gender: masculine, etymology: after the Italian cardinal Roberto Bellarmino, type species by original designation: Bellarminuspugnax); Penny 1977: 34 (species list); Penny 1982: 212–213 (synonymy); Oswald and Penny 1991: 11, 45 (synonymy); Ohl 2004: 157 (synonymy).

#### Diagnosis.

The genus *Nolima* can be distinguished by the following combination of characters (character states in parentheses are generally exhibited by other mantispid genera): a) Sc comes in contact with C near the middle of costal margin and distal to the base of pterostigma on the forewing (at apex of 2/3 of costal margin and proximal to pterostigma), b) M diverging from R distal to 1m-cu on the forewing (proximal to 1m-cu), c) abdominal terga and sterna or only terga of the male with circular or polygonal structures, respectively, d) male mediuncus apex strongly projecting posteriorly and deeply bifid (shallowly indented), and e) female spermatheca with accessory gland (generally without accessory gland, but if present then associated to copulatory bursa, e.g., species of *Calomantispa*).

#### Description.

*General*. Coloration pale yellow, with dark brown pigmentation as stripes or marks in specific areas (detailed in the text below).

*Head*. Hypognathous. Vertex with a rhomboid protuberance covering nearly its entire area; vertex marking M-shaped, extending behind antennal sockets, where can be bifurcated, if bifurcated then one branch extends posteriorly, parallel to anterior ocular margin, additional branch generally extends anteriorly on frontogenal furrow, or extends on frontogenal and epistomal furrows; vertex with a pair of irregular marks originating posteromedially, extending anteriorly along the coronal suture, then angled at 45° toward anterior ocular margin, reaching the rhomboid protuberance, sometimes converging with upper part of M-shaped mark. Frons generally with pair of semicircular marks. Clypeus and labrum, each sometimes with a medial semicircular mark. Antennal flagellomeres dark brown, as long as wide in basal third of flagellum, twice as long as wide in distal two thirds in frontal view. Mandibles with pigmentation on inner and outer edges.

**Figure 4.**
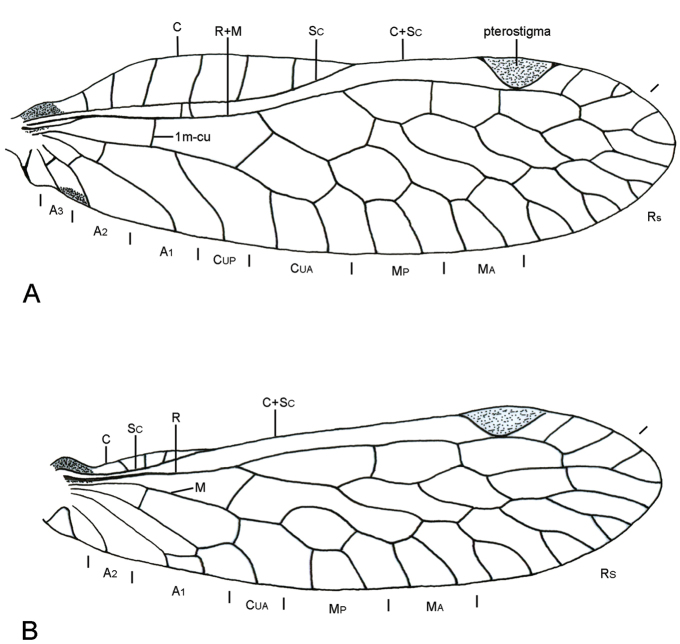
Wing venation of *Nolimapinal*. **A** forewing **B** hindwing.

*Thorax*. Prothorax straight in lateral view, with pigmentation, bristle-bearing chalazae on pronotum and anterolateral and anteroventral areas, a pair of pale spots anterolaterally in dorsal view. Mesothorax with conspicuous mesoscutal and scutoscutellar sutures; scutum generally with two longitudinal stripes anterior to suture and four posterior to suture, two medial and two lateral; scutellum with color pattern variable; pleural area generally with pigmentation. Metathorax with mesoscutal suture obsolete, scutoscutellar suture conspicuous; scutum generally with an M-shaped mark medially, a longitudinal stripe on each side of medial mark. Forecoxa with bristle-bearing chalazae. Forefemur with dorsal margin slightly convex, midsection in dorsal view approximately twice as wide as apex; longitudinal row of spines on ventral side weakly compressed laterally; tibia arched, two thirds as long as femur, with ventral carina; first tarsomere more than twice as long as second. Middle and hindleg not modified, finely and evenly setose. Forewing (Fig. 4A) with costal margin convex above costal cells, almost straight to distal margin of pterostigma; Sc fusing with C distally, above Rs stem; pterostigma semicircular, reddish-brown, no hyaline space between pterostigma and R_1_; M free basally, diverging from R distal to 1m-cu; 1m-cu slightly inclined; Cu branching reduced. Hindwing (Fig. 4B) with costal margin concave proximally and convex distally above costal cell, almost straight to distal margin of pterostigma; Sc fusing with C posterior to Rs stem; M not fused with R; CuP absent.

**Figure 5.**
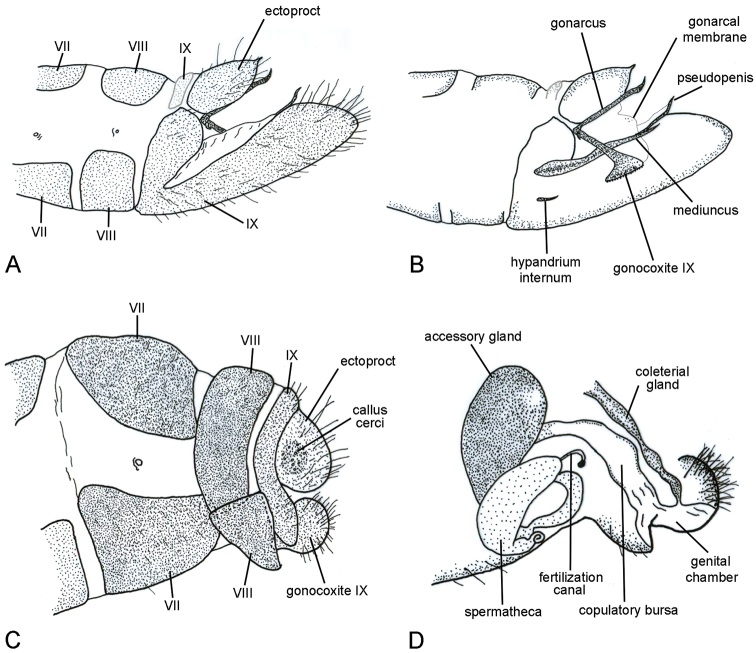
Last abdominal segments of *Nolimavictor*, lateral. **A** male external morphology **B** male internal morphology **C** female external morphology **D** female internal morphology.

*Abdomen*, *male* (Fig. 5A, B). Smaller than wing length at rest; terga and sterna I–VIII with circular structures barely touching each other (specially along midline) or terga I–VIII with polygonal structures in close contact to each other; terga and sterna I–VIII unfused laterally; tergum IX inconspicuous, narrow, almost reaching base of sternum IX; sternum IX elongate, posteriorly projected, scoop-like, with apodeme along basal margin. Ectoprocts with dorsal margin straight to strongly convex in lateral view, arched apodeme along basal margin, in dorsal view; ectoprocts fused dorsally, apex bilobed in dorsal view; apex of ectoprocts with microsetose membranous area between lobes, variably sclerotized; callus cerci not protuberant, obsolete. Gonarcus broadly or narrowly rounded in dorsal view, strongly sclerotized, apical process extending posterodorsally; gonarcal membrane with small tubercles dorsolaterally; gonarcus and gonocoxite IX associated basally, generally with laterally compressed apodemes extending anteriorly. Gonocoxite IX with posteroventrally inclined T-shape, small spines on apical and posteroapical surfaces. Mediuncus with obsolete to well-developed oval-shaped base, bifid apically; mediuncus apical processes strongly produced posteriorly, flanking pseudopenis. Pseudopenis sclerotized, lanceolate, produced further posteriorly than mediuncus processes. Hypandrium internum triangular in ventral view, longitudinal keel along midline.

*Abdomen*, *female* (Fig. 5C, D). Size similar to male; terga and sterna I–VIII without circular or polygonal structures; terga and sterna I–VII unfused laterally; tergum VIII narrow, ventrally produced, in contact with sternum VIII forming a ring; sternum VIII posteriorly produced, covering gonapophyses IX; tergum IX narrow, ventrally produced, not fused ventrally; sternum IX absent. Ectoprocts with margin convex in lateral view, apodeme along basal margin; ectoprocts fused dorsally, apex bilobed in dorsal view; apex of ectoprocts with membranous area between lobes; callus cerci not protuberant, conspicuous. Gonapophyses IX sclerotized, concave. Gonocoxite IX ovoid in lateral view, smaller than ectoproct. Genital chamber a membranous sac with several folds, located from posterior edge of sternum VIII to medial part of gonocoxite IX. Colleterial gland emerging from dorsal part of genital chamber, extending anterodorsally. Copulatory bursa dorsoventrally flattened, strongly sclerotized, narrowing anteriorly. Spermatheca lightly sclerotized, diverticulum in first third, with ovoid accessory gland. Fertilization canal long, narrow, apex bulbous.

#### Distribution.

This genus is endemic to the New World, ranging from southwestern United States to Costa Rica (Ohl 2004), including Guatemala, Honduras, and Mexico. Based on material examined, the species *N.pinal* and *N.victor* inhabit mountainous regions above 1500 m, primarily in areas with oak (*Quercus*) and pine (*Pinus*) vegetation. *Nolimainfensa* and *Nolimacostaricensis* sp. nov. occur in more tropical latitudes, from lowlands to mid-elevations.

#### Biology and natural history.

Little is known about this topic; the available information is related to the cytogenetics and larval diet of *Nolimapinal*.

#### Etymology.

The word *Nolima* is an anagram of Molina, in honor of Luis de Molina (1535–1600), a Jesuit priest who was born in the city of Cuenca, Spain (Navás 1914). The gender of this genus is considered feminine (Ohl 2004, JD Oswald, Texas A&M University, pers. comm.).

### Key to species of *Nolima* Navás

Most reliable diagnostic features are related to the external and internal genitalic morphology of males. Other traits (e.g., pigmentation, chalazae) are instructive for males and females but reliance on them alone may lead to misidentification.

**Table:** 

1	Forecoxa with bristle-bearing chalazae on ventral, lateral (basally), and dorsal surfaces; chalazae bases generally surrounded with dark brown pigmentation (Figs 9C, 10C)	**2**
–	Forecoxa with bristle-bearing chalazae only on ventral surface; chalazae bases not pigmented (Figs 6C, 8C)	**3**
2	Forefemur dorsal surface with conspicuous dark brown circular marks around bases of chalazae (Fig. 10C); prothorax ventral surface with dark brown longitudinal stripe; male abdominal terga I–VIII with polygonal structures (Fig. 10D); male ectoprocts with membrane between apices sclerotized (Fig. 10F, G); male ectoprocts with dorsal margin straight in lateral view (Fig. 10F)	***Nolimavictor* Navás**
–	Forefemur dorsal surface without dark brown marks around bases of chalazae (Fig. 9C); prothorax ventral surface without longitudinal stripe; male abdominal terga and sterna I–VIII with circular structures (Fig. 9D); male ectoprocts with membrane between apices generally not sclerotized (Fig. 9F, G); male ectoprocts with dorsal margin slightly convex in lateral view (Fig. 9F)	***Nolimapinal* Rehn**
3	Male ectoprocts with dorsal margin strongly convex in lateral view (Fig. 8F); male ectoprocts each with a cluster of long bristles anteromedially (Fig. 8F, G); pseudopenis conspicuously narrowing apically (Fig. 8I)	***Nolimainfensa* Navás**
–	Male ectoprocts with dorsal margin slightly convex in lateral view (Fig. 6F); male ectoprocts with fine setae evenly arranged over entire surface (Fig. 6F, G); pseudopenis not narrowing apically (Fig. 6I)	***Nolimacostaricensis* sp. nov.**

### Nolima costaricensis

Taxon classificationAnimaliaNeuropteraMantispidae

Reynoso & Contreras sp. nov.

http://zoobank.org/69F950F4-D1E8-472C-9ACC-C538FCDF0688

Figs 6 , 7

#### Diagnosis.

**Figure 6.**
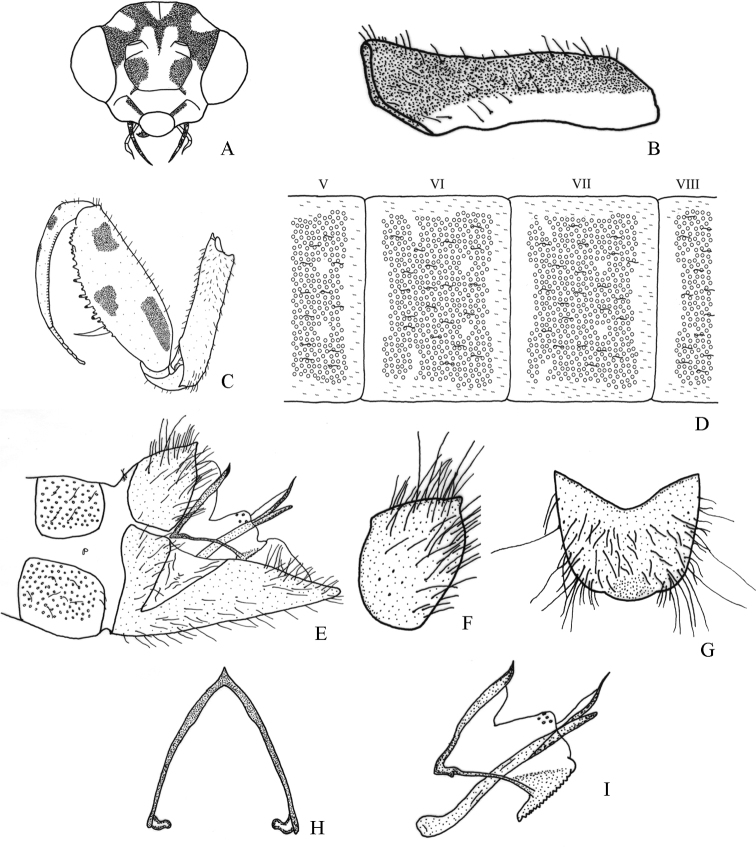
Structures of the male of *Nolimacostaricensis* sp. nov. **A** head, frontal **B** prothorax, lateral **C** left foreleg, lateral **D** abdominal terga V–VIII, dorsal **E** external terminalia, lateral **F** left ectoproct, lateral **G** ectoprocts, dorsal **H** gonarcus, dorsal **I** internal terminalia, lateral.

It differs from other species in the genus as follows: a) male sterna I–VIII with circular structures only laterally (Fig. 6E), b) male ectoprocts with membrane between apices not sclerotized, c) male ectoprocts with dorsal margin slightly convex (Fig. 6E, F), d) male ectoprocts with scattered long and short setae (Fig. 6E-G), e) gonarcus narrowly rounded (Fig. 6H), and f) pseudopenis not slender apically (Fig. 6I).

#### Note.

This new species is described based on a single male specimen collected in southeastern Costa Rica, which unfortunately had lost pigmentation; therefore we were not able to specifically evaluate some of the characteristic markings.

#### Description.

Male. *Head*. Vertex with M-shaped mark with lower arms getting wider towards anterior ocular margin (Fig. 6A); vertex irregular marks that originate posteromedially converging with upper part of M-shaped mark (Fig. 6A). Frons with a pair of large irregular marks laterally (Fig. 6A). Antennae 34-segmented; scape with indistinct pigmentation on posterior surface; pedicel with pigmentation on posterior surface.

*Thorax*. Prothorax with pigmentation on entire surface of pronotum (Fig. 6B). Forecoxa with bristle-bearing chalazae only on ventral surface, fine dark setae on most of remaining surface (Fig. 6C). Forefemur with three marks on lateral surface (Fig. 6C), mesal and dorsal surfaces without marks. Foretibia with two small dorsolateral marks on basal half (Fig. 6C). Middle and hind leg with fine dark setae.

*Abdomen*. Terga and lateral surface of sterna I–VIII with circular structures, not in contact to each other (Fig. 6D), microsetae in space between circular structures. Sternum IX with setae on entire surface, apex narrowly rounded in lateral view (Fig. 6E). Ectoprocts with dorsal margin slightly convex in lateral view; long and short setae scattered (Fig. 6F, G); membrane between apexes of ectoprocts not sclerotized, posteriorly produced (Fig. 6F), broadly rounded in dorsal view (Fig. 6G); basal apodeme of ectoprocts narrow, slightly sclerotized (Fig. 6G). Callus cerci obsolete. Gonarcus frail, narrowly rounded (Fig. 6H). Gonocoxite IX with base almost straight (Fig. 6I). Pseudopenis not slender apically (Fig. 6I).

#### Variation.

It could not be assessed because only the holotype specimen is known.

#### Biology and natural history.

Based on the collecting datum from the single specimen examined, adults of the species may be active during spring.

#### Etymology.

The species name is dedicated to Costa Rica, the only country from which this species is currently known.

#### Repository.

The holotype is housed at the INBIO.

#### Type locality.

Costa Rica: Puntarenas, Parque Internacional La Amistad, Sector Altamira.

#### Distribution.

**Figure 7.**
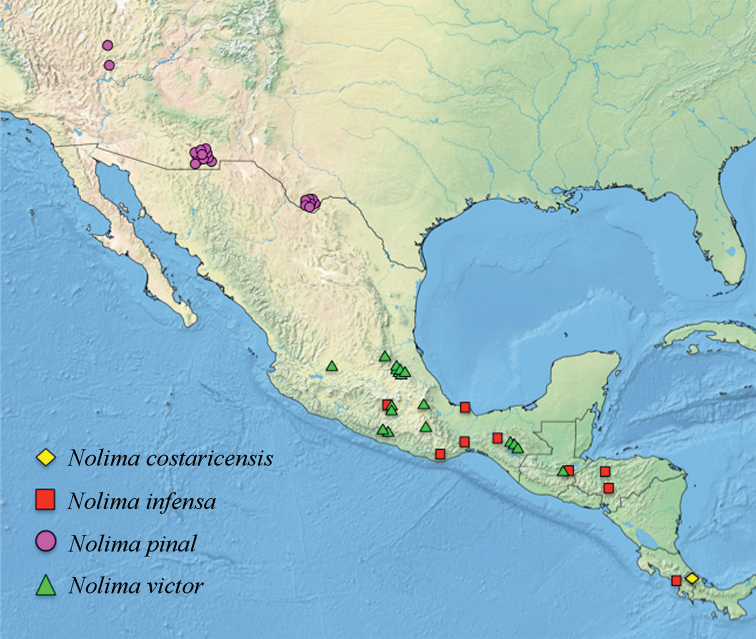
Distribution of the species in the genus *Nolima*.

This species is only known from its type locality, which is in the southeastern part of Costa Rica (Fig. 7), on the Talamanca range (1300–1400 meters). Because of the extension of the Talamanca range, it is likely the species is also distributed in Panama.

#### Type material examined.

HOLOTYPE ♂ (by present designation): **COSTA RICA**: **Puntarenas**: P[arque] I[nternacional] La Amistad, Sector Altamira, Send[ero] Gigantes del Bosque, 1300–1400 m, 13-IV–14-V-2005, R. González, T[ram]p[a] Malaise, LS331300 571500 #83526, INB0004129281, INBIOCRI, Costa Rica (INBIO).

### Nolima infensa

Taxon classificationAnimaliaNeuropteraMantispidae

Navás, 1924

Figs 3A , 7 , 8

Nolima infensus Navás, 1924: 61–62 (original description); Penny 1977: 36 (species list); Penny 1982: 213 (illustration); Henry et al. 1992: 449 (species list); Penny 1998: 212 (species list); Hoffman 2002: 252, 420–423 (species list, diagnosis, illustrations); Reynoso-Velasco and Contreras-Ramos 2010: 271–272 (species list, distribution).
Nolima infensa Navás: Ohl 2004: 158 (species list, correction of specific epithet original misspelling); Cancino-López et al. 2015: 202–203, 207–208 (species list, distribution, photo, systematics).

#### Diagnosis.

**Figure 8.**
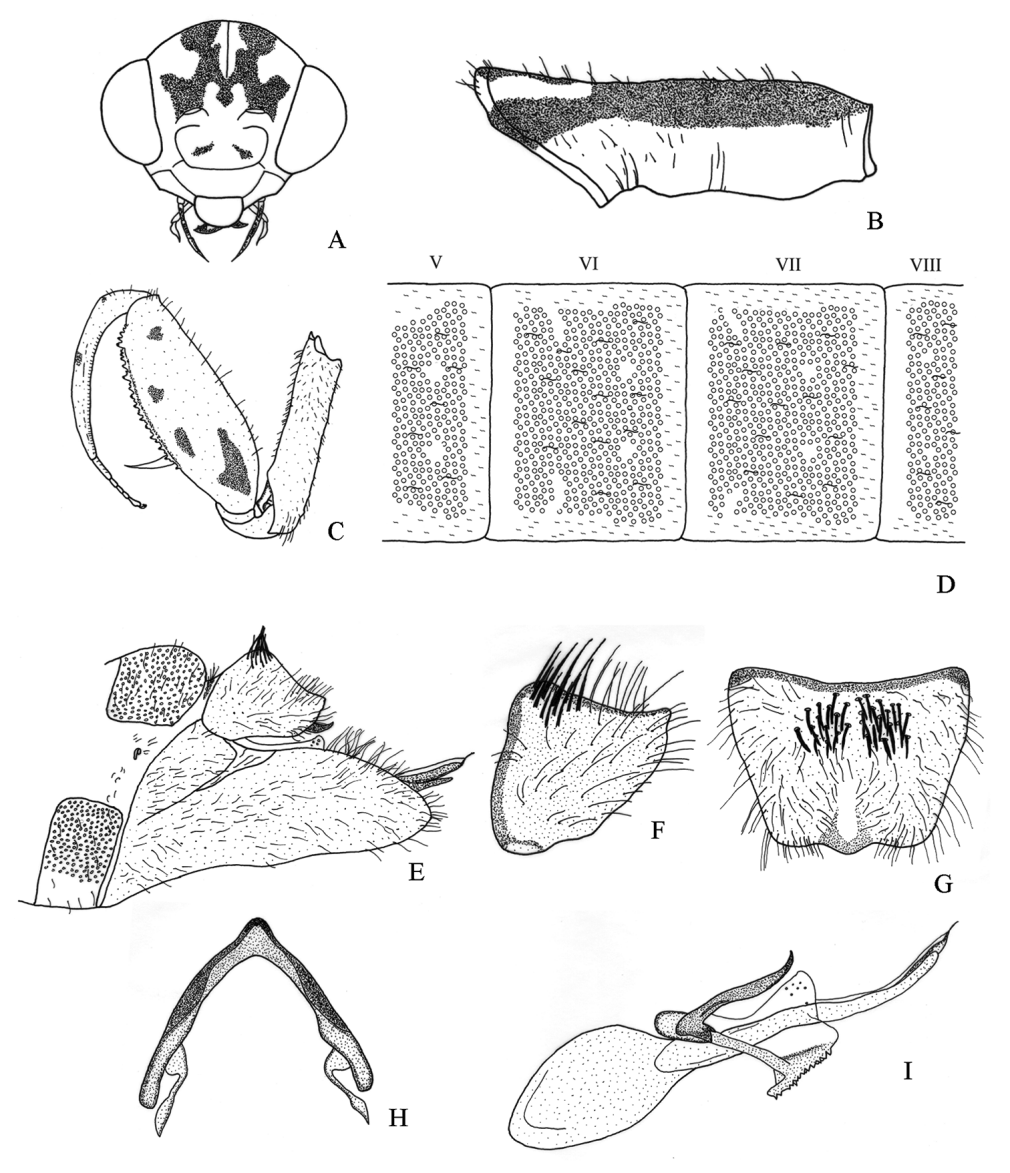
Structures of the male of *Nolimainfensa*. **A** head, frontal **B** prothorax, lateral **C** left foreleg, lateral **D** abdominal terga V–VIII, dorsal **E** external terminalia, lateral **F** left ectoproct, lateral **G** ectoprocts, dorsal **H** gonarcus, dorsal **I** internal terminalia, lateral.

It differs from other *Nolima* species as follows: a) male sterna I–VIII with circular structures only laterally (Fig. 8E), b) male ectoprocts with membrane between apices sclerotized, c) male ectoprocts with dorsal margin strongly convex (Fig. 8E, F), d) male ectoprocts each with cluster of long bristles anteromedially (Fig. 8E–G), e) gonarcus narrowly rounded (Fig. 8H), and f) pseudopenis slender apically (Fig. 8I).

#### Note.

This species was described based on a single female specimen collected in Costa Rica; Navás (1924) stated this species was similar to *N.victor*.

#### Description.

Male. *Head*. Vertex with M-shaped mark not bifurcated behind antennal sockets (Fig. 8A); vertex irregular marks that originate posteromedially converging with upper part of M-shaped mark (Fig. 8A). Frons with a pair of small irregular marks (Fig. 8A). Antennae 39 to 46-segmented; scape with longitudinal ovoid mark on posterior surface, pigmentation on distal margin; pedicel with pigmentation on posterior surface.

*Thorax*. Prothorax with pigmentation on pronotum, except anterolateral pale yellow mark on each side of midline (Fig. 8B). Forecoxa with bristle-bearing chalazae only on ventral surface, fine pale yellow setae on most of remaining surface (Fig. 8C). Forefemur with four marks on lateral surface (Fig. 8C), mesal and dorsal surfaces without marks. Foretibia with small dorsolateral mark medially (Fig. 8C). Mesopleuron generally pale yellow. Metapleuron with pigmentation on anepimeron, katepimeron, and meron. Middle and hind legs with fine pale yellow setae.

*Abdomen*. Terga and lateral surface of sterna I–VIII with circular structures, not in contact to each other (Fig. 8D), microsetae in space between circular structures. Sternum IX with setae on entire surface, apex broadly rounded in lateral view (Fig. 8E). Ectoprocts with dorsal margin strongly convex in lateral view; long bristles arranged in two clusters anteromedially (Fig. 8F, G); membrane between apexes of ectoprocts sclerotized, posteriorly produced (Fig. 8F), narrowly rounded in dorsal view (Fig. 8G); basal apodeme of ectoprocts broad, strongly sclerotized (Fig. 8G). Callus cerci obsolete. Gonarcus robust, narrowly rounded (Fig. 8H). Gonocoxite IX with base almost straight (Fig. 8I). Pseudopenis conspicuously slender apically (Fig. 8I).

Female. Pigmentation and setation generally same as for male.

#### Variation.

In both sexes, the pair of marks on the frons may be absent. An irregular mark may be present on the clypeus. The pigmentation on the forefemur may be absent. Specimens may also present pigmentation on the mesopleural katepisternum and anepimeron, on the metapleural anepisternum and katepisternum, or lack pigmentation on the pteropleural area. The dorsal margin of male ectoprocts may be only slightly convex in lateral view.

#### Biology and natural history.

Based on collecting data, adults of this species may be found active from May through August.

#### Etymology.

Navás (1924) did not specify the etymology of the species name. The specific epithet *infensus* is a Latin adjective meaning hostile or annoyed.

#### Repository.

The holotype is housed at the MNHN.

#### Type locality.

Costa Rica.

#### Distribution.

This species is distributed from central Mexico (Chiapas, Morelos, Oaxaca, Veracruz) south to Costa Rica (Puntarenas), including Guatemala (Zacapa) and Honduras (Comayagua, Yoro) (Fig. 7). Based on the material examined, elevation records (*n* = 4) range from 396 to 1,500 meters. Reported here are the first records of the species from Guatemala and Honduras. A male specimen of *N.infensa* from FSCA indicates it was collected in Florida (United States). As *Nolima* is distributed in the southwestern United States and considering that *N.pinal* is the sole species present in that area, the record from Florida is considered erroneous. Also, a female specimen at the NHMUK indicates it was collected in Guyana, South America. The specimen exhibits similar features to those of *N.infensa*, yet male specimens are required to confirm the species identification. This record is considered dubious based on the fact that no other *Nolima* specimens have been reported from nearby countries such as Colombia, where the fauna of Mantispidae has been recently studied (Ardila-Camacho and García 2015, Ardila-Camacho et al. 2018).

#### Published records.

Costa Rica; México: Morelos, Oaxaca (Navás 1924, Penny 1977, Henry et al. 1992, Ohl 2014, Reynoso-Velasco and Contreras-Ramos 2010, Cancino-López et al. 2015).

#### Type material examined.

HOLOTYPE ♀ (by monotypy): **COSTA RICA**: *Nolimainfensus* Nav. P. Navás S.J. det [1^st^ label], TYPE [2^nd^ label], Museum Paris, Costa Rica, Paul Serre 192 [3^rd^ label]. Microvial with last abdominal segments of the holotype in glycerine, pinned next to specimen: HOLOTYPE *Nolimainfensus* Navás ♀, Genitalia in Glycerin BEARD [single label] (MNHN).

#### Additional material examined.

**COSTA RICA: Puntarenas**: Las Alturas, 1500 m, 22-V-1992, F. Andrews & A. Gilbert, *Nolimainfensus* det. N. Penny (1♀, 1 adult without abdomen CAS). **GUATEMALA: Zacapa**: 12–14 km S San Lorenzo, 3-VI-1989, J. Wappes (1♂, 2♀ TAMU). [**GUYANA: East Berbice-Corentyne**]: British Guiana, New River, boundary mark 82, 1300 ft, 12-V-1938, C.[A.] Hudson (1♀ NHMUK). **HONDURAS: Comayagua**: Rancho Chiquito, Km 62, 2800 ft, 7-VI-1964, Blanton et al., blacklight trap (1♂ FSCA); **Yoro**: Pico Pijol, 22-VII-2001, R. Turnbow, mercury vapor light (1♂, 1♀ FSCA). **MEXICO: Chiapas**: [Ocozocoautla de Espinosa], Parque Laguna Bélgica, 2-VI-1991, B. Ratcliffe et al. (1♂ CASC); **Morelos**: [Mpio. Amacuzac], Huajintlán, carr. Amacuzac, 18°36'06"N, 99°25'19"W, 925 m, 4-VII-2005, H. Brailovsky & E. Barrera (1♀ CNIN); **Oaxaca**: [Mpio. Candelaria Loxicha], Portillo del Rayo, 3–4-VI-1987, L. Cervantes (1♂, 1♀ CNIN); [Mpio. Asunción Ixtaltepec], 12 mi S Chivela, 18-VIII-1959, L. Stange & A. Menke (1♀ FSCA); same but / ♂ genitalia close to my specimen ex [from] Oakland Park, Fla., leg. C.F. Dowling / not *Nolimapinal* E. MacLeod, 7-X-1979 (1♂ FSCA); **Veracruz**: [Mpio. Catemaco], Coyame, Lake Catemaco, 2-VII-1963, R.E. Woodruff, blacklight trap (1♀ FSCA). **UNITED STATES: Florida**: Broward Co., Oakland Park, [no day]-IV-1964, C.F. Dowling, at light (1♂ FSCA) [probably erroneous locality data].

### Nolima pinal

Taxon classificationAnimaliaNeuropteraMantispidae

Rehn, 1939

Figs 4 , 7 , 9

Nolima pinal Rehn, 1939: 256–259, 263 (key, original description); Hughes-Schrader 1979: 10–11 (cytogenetics); MacLeod and Redborg 1982: 38–41 (biology, photos); Lambkin 1986a: 3, 21 (species list, systematics); Willman 1990: 263 (illustration); Penny et al. 1997: 73 (species list); Ohl 2004: 158 (species list); Liu et al. 2015: 185, 200, 204 (species list, illustration, systematics); Winterton et al. 2018: 342, 344 (systematics).
Nolima dine Rehn, 1939: 256–257, 261–263 (key, original description); Penny et al. 1997: 73 (species list); Ohl 2004: 157 (species list) (new synonym).
Nolima kantsi Rehn, 1939: 256–257, 260–262 (key, original description); Penny et al. 1997: 73 (species list); Ohl 2004: 158 (species list) (new synonym).

#### Diagnosis.

**Figure 9.**
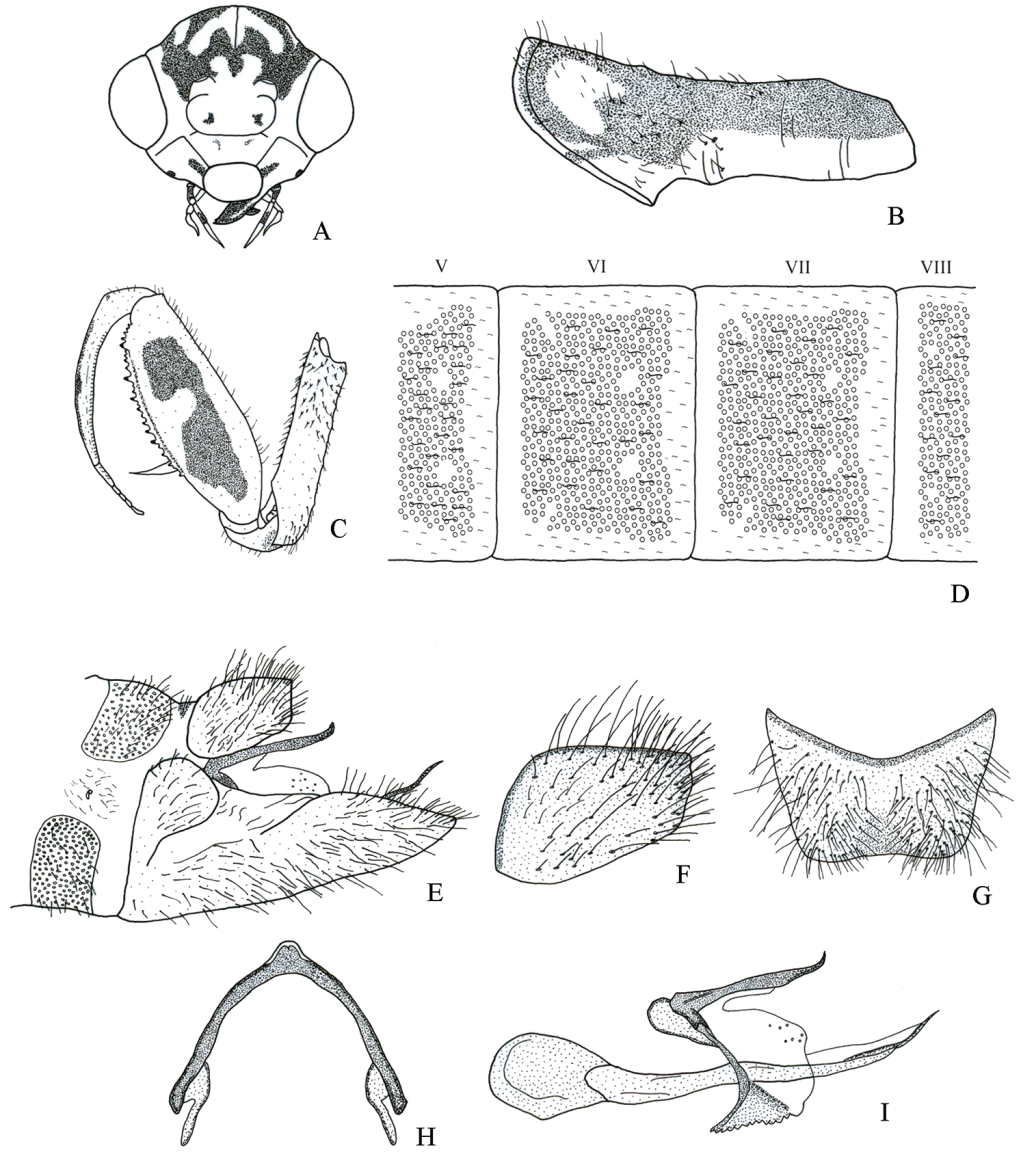
Structures of the male of *Nolimapinal*. **A** head, frontal **B** prothorax, lateral **C** left foreleg, lateral **D** abdominal terga V–VIII, dorsal **E** external terminalia, lateral **F** left ectoproct, lateral **G** ectoprocts, dorsal **H** gonarcus, dorsal **I** internal terminalia, lateral.

It differs from other species in the genus as follows: a) male sterna I–VIII with circular structures on nearly the entire surface (Fig. 9E), b) male ectoprocts with membrane between apexes not sclerotized, c) male ectoprocts with dorsal margin slightly convex (Fig. 9E, F), d) male ectoprocts with scattered long setae (Fig. 9E–G), e) gonarcus broadly rounded (Fig. 9H), and f) pseudopenis not slender apically (Fig. 9I).

#### Notes.

*Nolimapinal* was described based on a single female specimen collected in Arizona, United States. In the original description the holotype was erroneously reported as a male specimen. Rehn (1939) stated that this species was similar to *N.praeliator*. The distinction between *N.pinal* and the other two species in the United States, which were also described based on females but erroneously reported as males in the original descriptions, was based mainly on the pigmentation pattern of the head, pronotum, mesonotum, and metanotum, as well as width of the pronotum. After the examination of the type specimens of the three species from the southwestern United States and the additional material available for this study, we found that the pigmentation pattern used to distinguish among the species was not consistent, thus its aid in the species delimitation was questionable. In addition, after the examination of the male genital structures from specimens in the entire species distribution (southwestern United States), including specimens from the previously unknown range in Nevada, we found that the structures exhibited sufficient similarity to be considered a single species. Thus we propose *N.dine* and *N.kantsi* to be junior synonyms of *N.pinal*. Even when the name *N.pinal* has no position precedence because is not the type species of the genus (see *N.victor* section), we chose *N.pinal* as the valid name for this species only because it was the first to be described in the work by Rehn (1939, p. 257).

#### Description.

Male. *Head*. Vertex with M-shaped mark bifurcated behind antennal sockets, one branch extending posteriorly parallel to anterior ocular margin, additional branch extending anteriorly on frontogenal furrow (Fig. 9A); vertex irregular marks that originate posteromedially converging basally with branch of bifurcation extending posteriorly (Fig. 9A). Frons with a pair of small irregular marks (Fig. 9A). Antennae 29 to 39-segmented; scape with narrow longitudinal mark on posterior surface; pedicel with pigmentation on posterior surface.

*Thorax*. Prothorax with pigmentation on pronotum, except narrow pale yellow longitudinal stripe along midline and anterolateral pale yellow mark on each side of midline (Fig. 9B). Forecoxa with bristle-bearing chalazae on ventral, lateral, and dorsal surfaces; pigmentation on chalazae bases (Fig. 9C). Forefemur with one large mark on lateral surface (Fig. 9C), mesal and dorsal surfaces without marks. Foretibia with long dorsal mark on basal 2/3. Meso- and Metapleuron with pigmentation on anepisternum, anepimeron, katepisternum, katepimeron, and meron. Middle and hind legs with dark setae.

*Abdomen*. Terga and sterna I–VIII with circular structures, not in contact to each other, microsetae in space between circular structures (Fig. 9D). Sternum IX with setae on entire surface, apex narrowly rounded in lateral view (Fig. 9E). Ectoprocts with dorsal margin slightly convex in lateral view; long setae scattered (Fig. 9F, G); membrane between apexes of ectoprocts not sclerotized, not posteriorly produced, concave in dorsal view (Fig. 9G); basal apodeme of ectoprocts broad, strongly sclerotized (Fig. 9G). Callus cerci obsolete. Gonarcus robust, broadly rounded (Fig. 9H). Gonocoxite IX with base slightly curved (Fig. 9I). Pseudopenis not slender apically (Fig. 9I).

Female. Pigmentation and setation generally same as for male, except the antennal scape, which presents pigmentation on entire posterior surface.

#### Variation.

The mark located on the frontogenal furrow sometimes extends ventrally onto the epistomal furrow, a feature more common in females. The clypeus may present a single irregular mark medially. The anterolateral pale yellow mark of the pronotum sometimes exhibits pigmentation medially, giving an appearance of two marks. The forefemur may present two marks on the lateral surface, a trait more common in females. Some females from Texas exhibited three marks on the lateral surface of the forefemur. Also, the forefemur may present an elongate mark on the first half of the mesal surface. Sometimes the foretibia presents three dorsal marks. The membrane between apexes of ectoprocts may be slightly sclerotized.

#### Biology and natural history.

The cytogenetics of 15 species of mantispids from 11 genera and three subfamilies has been studied to date (Hughes-Schrader 1969, 1979); among these species, *N.pinal* has the lowest number of chromosomes. Its chromosomal complement consists of seven pairs of autosomes and one pair of sex chromosomes, XX (female) and XY (male). Under experimental conditions (Macleod and Redborg 1982), larvae of *N.pinal* were able to feed on a large variety of immature and adult insects and spiders, therefore it has been suggested the species is a generalist. In contrast, a certain degree of prey specialization has been documented for other mantispids (Parker and Stange 1965, Werner and Butler 1965, Redborg 1998). Mantispines are hypermetamorphic. The first instar is active and usually campodeiform, while later instars are vermiform or scarabaeiform and little active (Triplehorn and Johnson 2005). In contrast, larvae of *N.pinal* are ambulatory in all three larval instars, although they require prey to be sedentary because of low capacity of larval movement. In the laboratory (T = 25 °C, photoperiod L:D = 16:8) *N.pinal* took 15 days to go through three larval instars (from eclosion to just before construction of the cocoon) and 2–3 weeks in the pupal stage (MacLeod and Redborg 1982). Based on material examined, adults of *Nolimapinal* may be found active from April through September, being more common in August.

#### Etymology

. Rehn (1939) named this species after the Pinal Coyotero Apache group, which inhabited the region around the Pinal Mountains in Arizona, United States.

#### Repository.

The holotype is housed at the MCZ.

#### Type locality.

United States: Arizona: Gila Co., Pinal Mountains.

#### Distribution.

This species is distributed in the southwestern United States (Fig. 7). The species was reported from Arizona in the original description, also as *N.dine*. In addition, the species was reported from Texas as *N.kantsi*. Herein, *N.pinal* is reported from Nevada for the first time. Given this southern distribution in the United States, it may be expected the species is also distributed in the northern Mexican states of Chihuahua, Coahuila, and Sonora. Based on the material examined, this species may be found in areas with oaks at elevations (*n* = 9) ranging from 1,509 to 1,753 meters.

#### Published records.

United States: Arizona, New Mexico, Texas (Rehn 1939, Penny et al. 1997, Ohl 2004).

#### Type material examined.

HOLOTYPE ♀ (by original designation): **UNITED STATES: Arizona**: [Gila Co.], base of Pinal M[oun]t[ain]s, Ariz. [1^st^ label, with antennal flagellum glued], Sep[tember], D.K. Duncan [2^nd^ label], Oak [3^rd^ label], M.C.Z. type 23645 [4^th^ label], *Nolimapinal* Rehn TYPE [5^th^ label], MCZ [6^th^ label]. Microvial with last abdominal segments of the holotype in glycerine, pinned next to specimen: *Nolimapinal* ♀, 28.I.1985, Genital prep. nr. Ragnar Hall 103 [single label] (MCZ). Extra label in Holotype’s unit tray: The holotype of *Nolimapinal* Rehn is a ♀, not a male as described by Rehn, 21-X-1966, R. Beard. HOLOTYPE ♀.

#### Type material of synonyms examined.

**UNITED STATES: Arizona**: [Pinal Co.], Peppersauce C[a]n[yon], Aug. 16, 1924 [1^st^ label], Santa Catalina Mts. [2^nd^ label], J.O. Martin Collector [3^rd^ label], *Nolimadine* Rehn TYPE [4^th^ label], California Academy of Sciences Type No. 4927 [5^th^ label] (♀ CAS). PARATYPES: [Pinal Co.], Peppersauce C[a]n[yon], Aug. 16, 1924, Santa Catalina Mts., J.O. Martin Collector, *Nolimadine* Rehn Allotype (1♂ CAS); [Pinal Co.], Peppersauce Canyon, Aug. 17, 1924, E.P. Van Duzee, *Nolimadine* Rehn Paratype (1♀ CAS). HOLOTYPE ♀. **UNITED STATES: Texas**: Brewster Co., Chisos Mts., July 16 1921 [1^st^ label], C.D. Duncan Collector [2^nd^ label], *Nolimakantsi* Rehn TYPE [3^rd^ label], California Academy of Sciences Type No. 4926 [4^th^ label] (♀ CAS).

#### Additional material examined.

**UNITED STATES: Arizona**: Cochise Co., Cave Creek Canyon, 3 mi W Portal, 31°53.023'N, 109°10.715'W, 5120 ft, 9-VIII-2000, A. Gilbert & N. Smith (1♀ ZMB); Cochise Co., Chiricahua M[oun]t[ain]s, Cave Creek Ranch, 4880 ft, 14-VIII-1966, D. Alsop et al., 15w UV light (1♂ NMNH); Cochise Co., Paradise Cemetery Area, 5700 ft, 17-VIII-1977, S. Schrader-K. & R. Cooper-E., UV light beneath *Quercus* (5♂ 4♀ TAMU); Cochise Co., Paradise Cemetery Area, 5700 ft, 17-VIII-1977, R. Cooper-E., swept from *Quercus* (1♂ SDMC; 2♀ TAMU); Cochise Co., Paradise Cemetery Area, 5700 ft, 19-VIII-1977, R. Cooper-E., swept from *Quercus* (1♀ SDMC; 4♂, 5♀, 1 adult without abdomen TAMU); Cochise Co., Pinery Canyon, 3 mi E of j[un]ct[ion] Ariz[ona] 181, 5440–5600 ft, 17-VIII-1966, R.G. Beard & C. Weidert, beating oaks (1♀ NHMUK; 1♀ TAMU); same but 25-VIII-1966 (1♀ ZMB); Cochise Co., Portal Cave-Creek Ranch, 4900 ft, 17-VIII-1977, K. Cooper, UV light in woods (1♂ TAMU); Cochise Co., Portal Ranger Station, 4950 ft, 5-VIII-1966, R.G. Beard & R.E. Dietz (1♂ CASC; 1♂ MCZ); same but *Nolima* ♀66-L, ♀ died 9-VIII, eggs laid 8-VIII hatched (1♀ MCZ); Cochise Co., Portal Ranger Station, 4950 ft, 5-VIII-1966, R.G. Beard & R.E. Dietz, *Nolima* ♀66-M, ♀ died 9-VIII, eggs laid 8-VIII hatched (1♀ MCZ); Cochise Co., Portal Ranger Station, 4950 ft, 5-VIII-1966, R.G. Beard & R.E. Dietz, *Nolima* ♀66-N, ♀ died 9-VIII, eggs laid 8-VIII hatched (1♀ MCZ); Cochise Co., Portal Ranger Station, 4950 ft, 7-VIII-1966, R.G. Beard, beaten from oak, *Nolima* ♀66-P, ♀ died 11-VIII, eggs laid 10-VIII hatched (1♀ MCZ); Cochise Co., Portal Ranger Station, 4950 ft, 9-VIII-1966, R.G. Beard, UV light (1♀ MCZ; 1♀ MNHN); Cochise Co., Portal Ranger Station, 4950 ft, 12-VIII-1966, R.G. Beard, UV light, *Nolima* ♀66-R, ♀ died 19-VIII, eggs laid 18-VIII hatched (1♀ TAMU); Cochise Co., Portal Ranger Station, 4950 ft, 12-VIII-1966, R.G. Beard, UV light, *Nolima* ♀66-S, ♀ died 19-VIII, eggs laid 18-VIII hatched (1♀ MCZ); Cochise Co., Portal Ranger Station, 4950 ft, 13-VIII-1966, R.G. Beard, beaten from oak (1♀ CASC); Cochise Co., Portal Ranger Station, 12-VIII-1999, at light, M. Ohl (2♀ ZMB); Cochise Co., Paradise, 20-VIII-1978, [no collector] (1♀ SDMC); Cochise Co., Douglas, 7-VIII-1980 (1♂ CASC); Cochise Co., 5 mi W Portal, S[outh] W[estern] R[esearch] S[tation], 5400 ft, 15-VIII-1969, [no collector] (1♀ CASC); Cochise Co., Lowell, 26-VIII-1964, G.H. Nelson, flying (1♂ FSCA); Cochise Co., Portal, 6 mi above S[outh] W[estern] Res[earch] Sta[tion], 24-VII-1969, G.H. Nelson, beating *Quercushypoleuca* (1♂ FSCA); Cochise Co., Portal, 2-IX-1974, H. & M. Townes (1♀ FSCA); same but 6-IX-1974 (1♀ FSCA); same but 23-VIII-1987 (1♂ FSCA); same but 29-VIII-1987 (1♀ (FSCA); [Cochise Co.], 5 mi W Portal, Chiricahua M[oun]t[ain]s, 18-VIII-1958, D.D. Linsdale (1♀ FSCA); [Maricopa Co.], Seven Springs Ranger Sta[tion], 20-IV-1938, S.E. Crumb (1♀ TAMU); **Nevada**: Clark Co., Cabin C[an]y[o]n, 36.663062N, 114.070060W, 21-V-2008, C.W. Irwin, Lindgren trap PPQ07 (1♀ CASC); Lincoln Co., Spring Valley, 38.025963N, 114.208495W, 30-VIII-2008, R.J. Little, Lindgren trap BB60 (1♂, 3♀ CASC); **New Mexico**: Hidalgo Co., Animas M[oun]t[ain]s, Double Adobe Ranch, 5500 ft, 15-VIII-1952, H.B. Leech & J.W. Green (1♀ TAMU); **Texas**: [Brewster Co.], Big Bend State Park, 12-VII-1941, B.E. White (1♀ CASC); Brewster Co., B[ig] B[end] N[ational] P[ark], Laguna Medows Tr[ai]l, 29°15'17"N, 103°18'23"W, 5500–5750 ft, 20-VII-2002, E.G. & C.M. Riley, beating (1♀ TAMU); Brewster Co., B[ig] B[end] N[ational] P[ark], The Basin, 29°16'14"N, 103°17'54"W, 5600 ft, 21-VI-2004, E.G. Riley, UV light (1♀ TAMU); Brewster Co., B[ig] B[end] N[ational] P[ark], n[ea]r Lost Mine Trail, 29°16'03"N, 103°17'22"W, 5750 ft, 6-VI-2006, E.G. Riley, UV light (1♂ TAMU); Brewster Co., B[ig] B[end] N[ational] P[ark], The Basin ar[ea], 29°16'05'N, 103°18'09'W, 5600 ft, 5–8-VI-2006, E.G. Riley, UV [light] (1♂, 1♀ TAMU); Brewster Co., Chisos M[oun]t[ain]s, Panther Pass, 6000 ft, 2-VI-1973, D.C. Ferguson (1♂ USNM); [Brewster Co.], Chisos M[oun]t[ain]s, Big Bend Park, 3-VII-1946, E.C. Van Dyke (2♀ CASC; 1♀ ZMB); same but 6-VII-1946 (1♀ CASC); [Brewster Co.], Chisos Mountains, Big Bend Park, 16-VII-1956, H. & A. Howden (1♀ MCZ); [Brewster Co.], Chisos Mountains, Big Bend Park, 1-V-1959, Howden & Becker, at light (1♀ MCZ); [Brewster Co.], Chisos Mountains, Big Bend Park, 3-V-1959, Howden & Becker, beaten gray oak (*Quercusgrisea*) (1♂ MCZ); [Brewster Co.], Chisos Mountains, Big Bend Park, 9-V-1959, Howden & Becker, beaten juniper (*Juniperus* sp.) (1♀ MCZ); [Brewster Co.], Chisos M[oun]t[ain]s, 26-VI-1961, D.J. & J.N. Knull (1♂, 2♀ MCZ); [Brewster Co.], Chisos M[oun]t[ain]s, 26-VI-1963 (1♀ SRSU); Davis M[oun]t[ain]s, 7-VII-1946, E.C. Van Dyke (1♂, 1♀ CAS; 2♂, 1♀ TAMU).

### Nolima victor

Taxon classificationAnimaliaNeuropteraMantispidae

Navás, 1914

**Figure 10.**
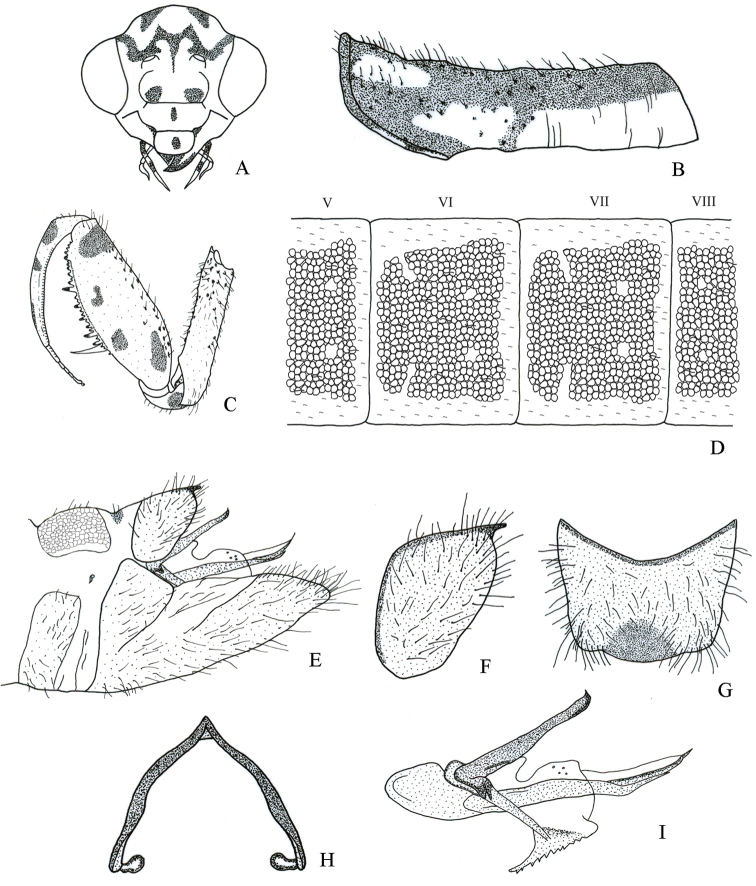
Structures of the male of *Nolimavictor*. **A** head, frontal **B** prothorax, lateral **C** left foreleg, lateral **D** abdominal terga V–VIII, dorsal **E** external terminalia, lateral **F** left ectoproct, lateral **G** ectoprocts, dorsal **H** gonarcus, dorsal **I** internal terminalia, lateral.

Figs 3B , 5 , 7 , 10

Nolima victor Navás, 1914: 101 (original description); Rehn 1939: 256–257 (systematics); Penny 1977: 36 (species list); Penny 1982: 213 (systematics); Oswald et al. 2002: 580 (species list, distribution); Ohl 2004: 158 (species list); Reynoso-Velasco and Contreras-Ramos 2008: 704–708 (species list, distribution, illustrations, as Nolima sp. 1); Reynoso-Velasco and Contreras-Ramos 2009: 710–711 (species list, distribution); Reynoso-Velasco and Contreras-Ramos 2010: 270–272 (species list, distribution); Cancino-López et al. 2015: 203, 205, 208 (species list, distribution, systematics).
Nolima praeliator Navás, 1914: 101–102 (original description); Rehn 1939: 256–257, 260–261 (systematics); Penny 1977: 36 (species list); Oswald et al. 2002: 580 (species list, distribution); Ohl 2004: 158 (species list); Reynoso-Velasco and Contreras-Ramos 2008: 708 (species list) Reynoso-Velasco and Contreras-Ramos 2010: 270 (distribution) (new synonym).
Nolima pugnax (Navás), 1914: 103 (original description); Henry et al. 1992: 449 (species list); Ohl 2004: 158 (species list) (new synonym).

#### Diagnosis.

It differs from other *Nolima* species as follows: a) male terga I–VIII with polygonal structures (Fig. 10D, E), b) male ectoprocts with membrane between apexes sclerotized, c) male ectoprocts with dorsal margin straight (Fig. 10E, F), d) male ectoprocts with scattered setae (Fig. 10E–G), e) gonarcus narrowly rounded (Fig. 10H), and f) pseudopenis not slender apically (Fig. 10I).

#### Notes.

The original description of *Nolimavictor* apparently was based on at least two specimens because in that work, Navás (1914) provided measurement ranges of the body and wings. However, during the first author’s visit to the NHMUK he only found one specimen, which is herein designated as the lectotype. In the same work, Navás (1914) described *N.praeliator* but reported only one measurement for the length of body and wings, suggesting the description was based on a single specimen, although Navás reported two specimens, one from Omiltemi and the other from Xucumanatlán. The latter was also reported as the type locality of *N.victor*. During the first author’s visit to the NHMUK he found only the specimen of *N.praeliator* collected in Omiltemi, which was clearly identified as the type. The specimen from Xucumanatlán cited in the original description of *N.praeliator* may have been the specimen used to describe *N.victor*. It is possible that Navás examined the two specimens from Xucumanatlán for the description of *N.victor* and erroneously cited one of them in the description of *N.praeliator*. The three type specimens mentioned in this section are females and as we have previously mentioned, the female genital structures are conserved and do not provide sufficient information for species identification. After examination and mainly based on characteristics of the forelegs (e.g., position of chalazae, pigmentation), we concluded the specimens were conspecific. Thus, we propose *N.praeliator* and *N.pugnax* as junior synonyms of *N.victor*. Even when the three species were described in the same work, the author clearly stated (Navás 1914, p. 21) that *N.victor* was the type species of the genus. Thus, this name has precedence and is the valid name for the species.

#### Description.

Male. *Head*. Vertex with M-shaped mark bifurcated behind antennal sockets, one branch extending posteriorly parallel to anterior ocular margin, additional branch not extending anteriorly (Fig. 10A); vertex irregular marks that originate posteromedially not converging with upper part of M-shaped mark (Fig. 10A). Frons with a pair of circular marks (Fig. 10A). Antennae 32 to 42-segmented; scape and pedicel without pigmentation on posterior surface.

*Thorax*. Prothorax with pigmentation on pronotum, except narrow pale yellow longitudinal stripe along midline and anterolateral pale yellow oval mark on each side of midline (Fig. 10B). Forecoxa with bristle-bearing chalazae on ventral, lateral, and dorsal surfaces; chalazae bases colored (Fig. 10C). Forefemur with four marks on lateral surface (Fig. 10C), mesal surface with circular mark; dorsal surface with dark brown circular marks at setal bases (Fig. 10C). Foretibia with basal, medial, and apical dorsolateral marks (Fig. 10C). Meso- and metapleuron with pigmentation on anepimeron, anepisternum, katepimeron, katepisternum, and meron. Middle and hind leg with dark setae.

*Abdomen*. Terga I–VIII with polygonal structures, in close contact to each other (Fig. 10D), inconspicuous microsetae along margin of polygonal structures. Sternum IX with setae on entire surface, apex narrowly rounded in lateral view (Fig. 10E). Ectoprocts with dorsal margin straight in lateral view; setae scattered (Fig. 10F, G); membrane between apexes of ectoprocts sclerotized, posteriorly produced (Fig. 10F), broadly rounded in dorsal view (Fig. 10G); basal apodeme of ectoprocts broad, strongly sclerotized (Fig. 10G). Callus cerci obsolete. Gonarcus robust, narrowly rounded (Fig. 10H). Gonocoxite IX with base almost straight (Fig. 10I). Pseudopenis not slender apically (Fig. 10I).

Female. Pigmentation and setation generally same as for male.

#### Variation.

Both sexes may exhibit a circular mark on clypeus and one on labrum. The pronotum may be yellowish, with pigmentation only on the chalazae. In females, the bifurcated M-shaped mark on the vertex may present the branch that extends anteriorly, on the frontogenal and epistomal furrows. The irregular marks that originate posteromedially on vertex may be fused to bifurcation of M-shaped mark that extends posteriorly. Also, the antennal scape may exhibit a small mark on the mesal surface and the pedicel may be pigmented on the posterior surface. Sometimes with small circular marks on entire surface of forefemur.

#### Biology and natural history.

Based on collecting data from material examined, adults of this species are active from February through October.

#### Etymology.

Navás did not specify the etymology of the species name. However, the specific epithet *victor* is a Latin adjective meaning victorious. According to this, the name could be read as “Molina victorious,” a phrase acclaimed by Father Molina’s adherents when in 1607 Pope Paul V decided not to condemn the ideas of Molinism.

#### Repository.

The lectotype is housed at the NHMUK.

#### Type locality.

México: Guerrero: Mpio. Chilpancingo de los Bravo, Xocomanatlán.

#### Distribution.

This species is distributed in Mexico (Chiapas, Guerrero, Hidalgo, Jalisco, Morelos, Oaxaca, Puebla, Querétaro) and Guatemala (Baja Verapaz) (Fig. 7). Elevation records of this species are the highest known for the genus, ranging from 2,134 to 2,775 meters. This species was previously reported from the Mexican state of Guerrero as *N.praeliator*. In addition, it was reported as *N.pugnax* from San Jerónimo, in the Guatemalan department of Baja Verapaz. Herein, the species is reported for the first time from the state of Puebla in central Mexico.

#### Published records.

Guatemala: Baja Verapaz; México: Chiapas, Guerrero, Hidalgo, Jalisco, Morelos, Oaxaca, Querétaro (Navás 1914, Penny 1977, Henry et al. 1992, Oswald et al. 2002, Ohl 2004, Reynoso-Velasco and Contreras-Ramos 2008, 2009, 2010, Cancino-López et al. 2015).

#### Type material examined.

LECTOTYPE ♀ (by present designation): **MEXICO: Guerrero**: [Mpio Chilpancingo de los Bravo], Xucumanatlan [Xocomanatlán], 7000 ft, July [no year], H.H. Smith [1^st^ label, with antennal flagellum glued], Godman-Salvin Collection 1913-214 [2^nd^ label], Typus [3^rd^ label], *Nolimavictor* ♀ Nav. Navás S.J. det. [4^th^ label], Genitalia prep. in vial on other pin made 20-V-1969, R.G. Beard # 1008 [5^th^ label], Type H.T. [6^th^ label]. Microvial with last abdominal segments of the holotype in glycerine, pinned next to specimen: HOLOTYPE ♀ *Nolimavictor* Navás 1909, ♀ Genitalia in glycerine [1^st^ label], R.G. Beard # 1008 Genitalia prep. of *Nolimavictor* Navás 1909 ♀ Holotype in glycerine [2^nd^ label] (NHMUK).

#### Type material of synonyms examined.

[**MEXICO]: Guerrero**: [Mpio Chilpancingo de los Bravo], Omilteme [Omiltemi], 8000 ft, Aug., H.H. Smith [1^st^ label], Godman-Salvin Collection 1913–214 [2^nd^ label], *Nolimapraeliator* Nav. Navás S.J. det. [3^rd^ label], Typus [4^th^ label], *Nolimavictor* Navás ♀ D. Reynoso-Velasco det. 2008 [5^th^ label], NHMUK 012502477 [6^th^ label] (NHMUK) [SYNTYPE ♀ of *N.victor*]. **GUATEMALA: [Baja Verapaz**]: San Geronimo [Jerónimo]. Champion [1^st^ label], Godman-Salvin Collection 1913–214 [2^nd^ label], *Bellarminuspugnax* Nav. Navás S.J. det. [3^rd^ label], Typus [4^th^ label], *Bellarminuspugnax* Nav., ♀ type, H.T. genital prep. made by Ragnar Hall 10.XI.1982 [5^th^ label], *Nolimavictor* Navás ♀ D. Reynoso-Velasco det. 2008 [6^th^ label], NHMUK 012502476 [7^th^ label].

#### Additional material examined.

**MÉXICO: Chiapas**: Hwy 199, 11 km NE San Cristóbal, 8000 ft, 25-V-1987, D.A. Rider et al. (1♀ TAMU); Mpio. Huixtlán [Huixtán], 2.4 km NE Chilil, camino a F[ray] Bartolomé, 23-V-1995, M. Girón (1♀ ECOSUR); 10 mi SE Teopisca, 20-VI-1965, Burke et al. (1♀ TAMU); **Hidalgo**: [Mpio.] Huasca [de Ocampo], R[an]cho Santa Elena, Manantial de Las Vigas, 2300 m, 21-V–3-VI-2003, Contreras-Ramos & Menchaca-Armenta, Malaise 2 (1♂ CNIN); same but 3-VI–19-VI-2003 (1♂ CNIN); [Mpio.] Huasca [de Ocampo], R[an]cho Santa Elena, Manantial de Las Vigas, 17-VI–3-VII-2003, Contreras-Ramos & Meléndez-Ordóñez, Malaise 1 (1♀ CNIN); [Mpio.] Huasca [de Ocampo], R[an]cho Santa Elena, Manantial de Las Vigas, 16-VII–19-VIII-2003, Contreras-Ramos, Malaise 1 (1♀ CNIN); [Mpio.] Huasca [de Ocampo], R[an]cho Santa Elena, Manantial de Las Vigas, 20°07'53.4"N, 98°31'38.5"W, 19-VIII–19-IX-2003, Contreras-Ramos & Menchaca-Armenta, Malaise 1 (1♀ CNIN); [Mpio.] Huasca [de Ocampo], R[an]cho Santa Elena, Manantial de Las Vigas, 20°07'53.4"N, 98°31'38.5"W, 2300 m, 5-IX–3-X-2005, Meléndez-Ordóñez & Reynoso-Velasco, Malaise 1 (2♀ CNIN); [Mpio.] Huasca [de Ocampo], R[an]cho Santa Elena, Manantial de Las Vigas, 20°07'52.2"N, 98°31'39"W, 2480 m, 3–31-X-2005, Contreras-Ramos et al., Malaise (1♂ CNIN); same but 23-II–23-III-2006 (1♀ CNIN); [Mpio. Mineral del Chico], P[arque] N[acional] El Chico, 20°11'18.7"N, 98°44'33.3"W, 2775 m, pine forest, 1-X–12-X-2002, J. Asiain & J. Márquez, pitfall trap (squid) (1♀ CNIN); **Jalisco**: Mpio. Degollado, La Sanguijuela, 14-VII-1995, R. Ayala (1♀ EBCH); **Morelos**: 8 km N Cuernavaca, Hwy 95, 5-IX-1982, C. O’Briend et al. (1 adult without abdomen CAS); **Oaxaca**: 8 mi SE Nochixtlán, 7500 ft, 13-VIII-1974, W. O’Brien et al. (2♀ CAS); **Puebla**: [Mpio. Nicolás Bravo], 4 miles east of Azumbilla, 22-VII-1984, Carroll et al. (1♀ TAMU); **Querétaro**: 4.5 km Carr[etera] La Lagunita-Tilaco, N 21 12 75, O 99 14 18, 27-II-1998, E. Barrera & G. Ortega (1♀ CNIN).

## Supplementary Material

XML Treatment for Nolima

XML Treatment for Nolima costaricensis

XML Treatment for Nolima infensa

XML Treatment for Nolima pinal

XML Treatment for Nolima victor
